# Characterization of Porcine Monocyte-Derived Macrophages Cultured in Serum-Reduced Medium

**DOI:** 10.3390/biology11101457

**Published:** 2022-10-04

**Authors:** Hana Štěpánová, Lenka Kavanová, Lenka Levá, Monika Vícenová, Kamil Šťastný, Martin Faldyna

**Affiliations:** Veterinary Research Institute, 62100 Brno, Czech Republic

**Keywords:** pig, porcine, monocyte-derived macrophages, serum reduction, in vitro

## Abstract

**Simple Summary:**

The aim of this study was to provide an improved in vitro model of porcine monocyte-derived macrophages (MDMs) based on reduced-serum culture. The partial or complete absence of serum in media is suitable for experimental studies focused on molecules that are naturally present in serum or in cultures used for subsequent protein purification. Moreover, the benefit of serum-free or reduced-serum supplements follows the current trend of reducing FBS use in biological research. In order to confirm that a reduced-serum MDM culture is suitable for in vitro functional studies of porcine macrophages, we assessed their morphology, yield after differentiation, surface marker expression, respiratory burst, and phagocytic activity, as well as the induction of cytokine gene expression.

**Abstract:**

The aim of this study was to establish a cell culture system for the generation of porcine monocyte-derived macrophages (MDMs) under reduced-serum conditions. Cultures based on either the Nu-Serum™ Growth Medium Supplement (NUS) or a conventional fetal bovine serum (FBS) were compared, which included the assessment of FBS from two different providers (FBS1 and FBS2). The data obtained confirmed the significant impact of culture conditions on in vitro-generated MDMs. The MDMs cultured under reduced-serum conditions showed increased levels of IL-1β and CD86 mRNA and a proinflammatory cytokine profile, characterized by the increased mRNA expression of IL-23p19, CXCL10, and CCL5. Phagocytic and respiratory burst activities were not adversely affected. Surprisingly, the difference between the two FBSs was much more pronounced than the effect of the reduced-serum supplement. The FBS1 culture conditions gave rise to macrophages with higher surface levels of CD14, CD16, and CD163, a lower CD80 mRNA expression, and an increased induction of IL-10 gene expression. In contrast, none of these trends were observed in macrophage cultures supplemented with FBS2. Instead, the FBS2 culture showed increased levels of IL-1b and CD86 mRNA. In conclusion, reduced-serum culture is a useful tool for in vitro porcine MDM generation, in line with the current research trend of reducing FBS use in biological research.

## 1. Introduction

Macrophages are a type of myeloid white blood cell with the unique ability to ingest and degrade foreign particles, microbes, and cellular debris. They play a critical role in immune response initialization and regulation by secreting a wide range of cytokines. Mature macrophages vary slightly in morphology and are named according to their tissue of residence [1]: e.g., Kupffer cells in the liver, microglia in the nervous system, pulmonary alveolar macrophages, peritoneal macrophages, etc.

Several types of in vitro models are used in macrophage research. They include permanent macrophage cell lines (the murine RAW 264.7 cell line being one of the best-known examples), primary macrophages isolated from tissue, or macrophages derived from blood monocytes. Permanent cell lines are easily accessible and “user-friendly” but are a slightly artificial model and become unstable through serial passage [2]. In contrast, primary macrophages carry their tissue-related biological properties with demanding and time-consuming isolation. Monocyte-derived macrophages (MDMs) represent a reasonable compromise, with a possible modulation and variation of biological features. For the most part, MDMs are the only in vitro model available for macrophage studies in research using nonlaboratory species, such as pets and wild or farm animals.

In pigs, the MDM model has been used for a large variety of studies, including testing of antibacterial [3,4] and antiviral [5,6,7] immune responses or testing preparations for clinical practice in both animals and humans [8,9]. The origin and methods for preparing and culturing porcine MDMs vary across different studies. Monocytes originating from blood [3,4] or bone marrow [10] have both been used. Purified CD14^+^ monocytes enriched by positive magnetic bead selection [3] or a mixture of mononuclear cells have been used as MDM precursors [4,10]. Monocytes differentiated from macrophages are only driven by plastic adherence [3,4] or in the presence of human/murine cytokines, such as M-CSF [10]. Culture media RPMI or D-MEM are supplemented with fetal bovine serum (FBS) [5,6,7], porcine serum [3,4,11], or autologous plasma [12]. Combinations of the abovementioned factors offer a wide spectrum of possible protocols for porcine MDM generation, which could result in phenotypic and functional heterogeneity.

Supplementation of the culture medium with FBS is still a common practice in biological research. However, ethical and animal welfare concerns have attracted global attention, leading to the reduced use of FBS and an increase in the use of alternatives to animal serum in culture media [13]. The first experiments with a serum-free culture of mammalian cells were established in the 1960s [14], and the advantages of serum-free supplements have been widely recognized in the decades that followed, including in terms of batch-to-batch uniformity, defined components, exclusion of potential virus contamination, and animal use reduction. This led to the development of a number of reduced-serum and serum-free media in recent decades [15]. These products offer FBS alternatives in conformity with the EU Directive 2010/63/EU on the protection of animals used for scientific purposes.

The aim of this study was to establish a cell culture system for the generation of porcine MDMs under reduced-serum conditions. Blood monocytes, which were obtained via a selection of CD14-positive cells, were used for the culture of high-purity MDMs. Several parameters, including morphology, yield, surface marker expression, cytokine profiles, and phagocytic and respiratory burst capacity, were assessed and a comparison with conventional FBS-containing culture was conducted.

## 2. Materials and Methods

### 2.1. Porcine Monocyte-Derived Macrophage Preparation and Culture

Porcine MDMs were prepared from purified CD14^+^ blood monocytes as previously described [3]. Briefly, blood was collected from the jugular vein of 3-month-old pigs. Animal care and use protocols were approved by the Ethical Committee of the Veterinary Research Institute according to guidelines set out in the *Animal Protection Act* and were subsequently approved by the Branch Commission for Animal Welfare of the Ministry of Agriculture of the Czech Republic (reference number 5050/2020-MZE-18134). Blood samples were heparinized using 25 IU/mL of sodium heparin (Zentiva, Prague, Czech Republic). Peripheral blood mononuclear cells (PBMCs) were isolated by gradient centrifugation using Histopaque-1077 (Sigma-Aldrich, St. Louis, MO, USA). Monocytes were enriched by positive magnetic bead selection (QuadroMACS™ cell separator, Miltenyi Biotec, BergischGladbach, Germany) using an anti-CD14 antibody (clone MIL2, AbD Serotec, Oxford, UK) and goat anti-mouse IgG microbeads together with LS separation columns (Miltenyi Biotec) according to the manufacturer’s recommendations. The average purity was checked by flow cytometry and was greater than 90%. Purified CD14^+^ cells were cultured in 24-well plates at a concentration of 5 × 10^5^ cells per well in 1 mL of D-MEM with antibiotics (100 IU/mL penicillin and 100 μg/mL streptomycin) and supplemented with 10% FSB1 (Diagnovum, Tilburg, The Netherlands; Superb, heat inactivated, collected in South America), FBS2 (HyClone, South America origin, GE Healthcare Life Sciences, Cramlington, UK), or Nu-Serum™ Growth Medium Supplement (Corning, Corning, NY, USA). Nu-Serum contains 25% of newborn calf serum along with a standardized, constant formulation of epidermal growth factor, endothelial cell growth supplement, insulin, transferrin, triiodothyronine, progesterone, estradiol 17-beta, testosterone, hydrocortisone, selenous acid, *o*-phosphorylethanolamine, glucose, amino acids, vitamins, and other trace elements and nutrients contained in Ham’s F12 medium base according to the certificate of analysis. All FBSs included in the experiments were heat-inactivated and filtered (0.2 µm), and all supplements were declared endotoxin-free. The cells were incubated for 5 days at 37 °C in 5% CO_2_ to differentiate into macrophages. The phenotypical and functional characterization of MDMs was performed as described below. For these purposes, the old medium was removed, the cells were washed with Hanks’ balanced salt solution without Ca^2+^ and Mg^2+^ (HBSS; Lonza, Basel, Switzerland), and HBSS with Ca^2+^ and Mg^2+^ (Lonza) was added without any other supplement. After 5 days of differentiation, the viable macrophage count was measured using the Cell Counting Kit-8 (CCK-8; Sigma-Aldrich) according to the manufacturer’s instructions. Cell morphology was assessed by light microscopy (Olympus IX51) with an Olympus LCAch 40×/0.55 RC3 objective using the Hoffman modulation contrast system.

### 2.2. Surface Marker Detection by Flow Cytometry

The phenotypical and functional characterization of MDMs was performed after 5 days of cultivation in different conditions. Firstly, surface markers, including CD14, CD16, CD163, and MHC II, were measured by flow cytometry. MDMs were detached from the plates by incubation at 4 °C with PBS containing 0.2% EDTA. The cells were washed once in a cell wash solution and stained with unconjugated primary antibodies. The primary mouse anti-pig antibodies were as follows: anti-CD14 (MIL2, IgG2b, Bio-Rad, Hercules, CA, USA), anti-CD16 (G7, IgG1, Bio-Rad), anti-CD163 (2A10/11, IgG1, Bio-Rad), and anti-MHC II (MSA3, IgG2a, WSU, Pullman, WA, USA). As secondary antibodies, IgG isotype-specific Alexa Fluor 488-conjugated goat anti-mouse antibodies were used (Thermo Fisher Scientific, Invitrogen, Waltham, MA, USA). Control samples were only stained with the secondary antibody. Flow cytometry was performed using an LSR Fortessa flow cytometer operated by Diva software, version 6.0 (Becton Dickinson, Franklin Lakes, NJ, USA). Doublets, which were defined by plotting the width against the area for forward scatter, and dead cells, which were stained with propidium iodide, were excluded from the analysis. The level of marker expression was evaluated as the median fluorescence intensity (MFI).

### 2.3. Chemiluminescence Assay

Respiratory burst was measured using chemiluminescence (CL). The assay was performed in Nunc-Immuno™ MicroWell™ 96-well polystyrene plates (Sigma-Aldrich). Purified CD14^+^ monocytes were seeded into the plate on the day of isolation at a concentration of 1 × 10^5^ cells per well in 0.2 mL of complete medium and cultured as described above. The old medium was removed immediately before measurement, the cells were washed with HBSS without calcium and magnesium (Lonza), and HBSS with calcium and magnesium was then added. Luminol derivative L-012 (Wako Chemicals GmbH, Neuss, Germany) was added to amplify the CL induced by the respiratory burst of stimulated cells. L-012 was diluted in HBSS to a final concentration of 0.15 mmol/L. CL was measured in cells stimulated by phorbol myristate acetate at a final concentration of 0.5 µg/mL (Sigma-Aldrich) and in non-stimulated cells (spontaneous chemiluminescence). The final volume of each well was 200 µL, and culture triplicates were included for all stimulations. Chemiluminescence was measured using a multi-detection microplate reader Synergy H1 (BioTek, Winooski, VT, USA) in kinetic mode for 2 h. The results were expressed as integrals of chemiluminescence intensity.

### 2.4. Phagocytic Activity Detection by Flow Cytometry

Phagocytic activity was measured by flow cytometry using fluorescently labeled bioparticles. A 5-day MDM culture in HBSS with Ca^2+^ and Mg^2+^ and without any medium supplements was incubated in the presence of Alexa Fluor 488-conjugated zymosan A bioparticles (Life Technologies, Carlsbad, CA, USA, Invitrogen) for 60 min at 37 °C. The cells were detached from the plates by incubating in 0.2% EDTA at 4 °C for 30 min. Propidium iodide was used to distinguish between live and dead cells. Flow cytometry was performed using an LSR Fortessa flow cytometer operated by Diva software (Becton Dickinson). A minimum of 50,000 events were acquired. Doublets were identified by plotting the width against the area for forward scatter and excluded. The percentage of Alexa Fluor 488-positive cells was evaluated from live single cells.

### 2.5. Cytokine Gene Expression Determination by Quantitative RT-PCR Analysis

Macrophages (0.5 × 10^6^ MDMs per well) were stimulated by nonopsonized zymosan (final concentration of 50 µg/mL; Sigma-Aldrich) for 4 h. Afterward, MDMs were lysed in 0.5 mL of TRI Reagent RT (Molecular Research Center, Cincinnati, OH, USA) and stored at −80 °C until RNA isolation. After phase separation using 4-bromoanisole, total RNA was purified using a NucleoSpin RNA Mini Kit (Macherey Nagel, Düren, Germany) according to the standard protocol. The quantity and quality of RNA were assessed spectrophotometrically, and RNA integrity was confirmed using agarose gel. mRNA was reverse-transcribed using a Luna Script RT SuperMix Kit (NEB) according to the manufacturer’s instructions. cDNA was stored at −20 °C and diluted 5× with RNAse-free water before use. qPCR reactions, with a total volume of 3 μL, were prepared using a Nanodrop II liquid dispenser (IDEX Health & Science LLC, Oak Harbor, WA, USA) and performed in triplicate on 384-well plates using a LightCycler 480 instrument (Roche, Basel, Switzerland) as follows: 0.5 μL of cDNA and 1.5 μL of Qiagen QuantiTect SYBR Green PCR MasterMix (Qiagen, Hilden, Germany) with 10 pmol of each gene set. Primers were adopted, as follows, from the indicated sources: IL-1β, IL-18, IL-23p19 [16], IL-10 [17], TNF-α [18], CCL2, CCL5, and CXCL10 [4]. In the variability NormFinder test [19] of the housekeeping genes, hypoxanthine phosphoribosyltransferase 1 (HPRT1) was selected as the most suitable reference gene among the tested samples and also served as a positive control. Assuming the primer efficiency ≥1.9 and normalized gene expression based on the quantification cycle, the Cq value [20] was calculated as 2^−(CqGENE−CqHPRT1)^ [21]. The specificity of amplification was confirmed in melting temperature analysis. Negative controls were set up for each part of the gene expression assessment. The normalized mRNA levels of the genes of interest are expressed in “HPRT units”.

### 2.6. Statistical Analysis

Data were analyzed using nonparametric Friedman test with Dunn’s multiple-comparison post hoc test; *p*-values < 0.05 were considered to indicate significant differences. Outliers were excluded from the analysis according to Grubbs test. Statistical analysis was performed and graphs were created using GraphPad prism software (GraphPad Software, version 5.04, San Diego, CA, USA). Furthermore, the experimental data were adjusted to the dataset X (*n* × *m*) with *n* = 17 variables (parameters) and *m* = 18 cases. All datasets were modified for use by multivariate statistical modeling with scaling. Missing data in several cases were replaced by robust nonparametric median estimates. Principal component analysis (PCA) was used for multivariate statistical analysis of the dataset and to evaluate relationships among variables. PCA data analysis was performed using STATISTICA, version 13.3 (TIBCO Software Inc., Palo Alto, CA, USA).

## 3. Results

### 3.1. Morphology and Yield of Porcine Monocyte-Derived Macrophages (MDMs) Generated In Vitro

**Figure 1:**
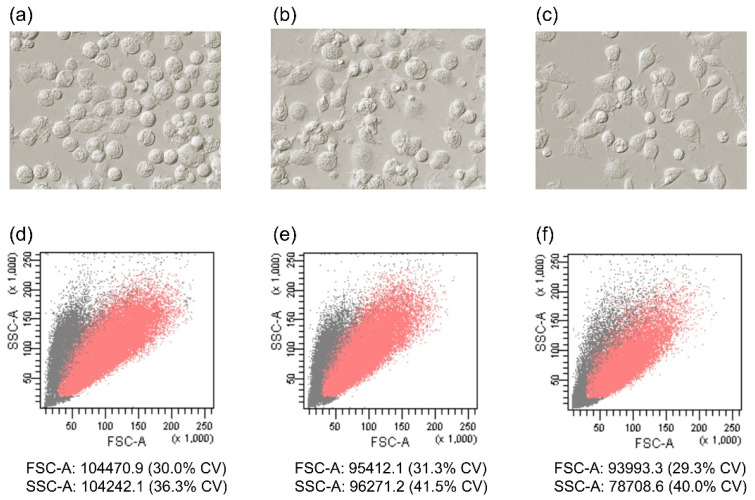
Morphology of monocyte-derived macrophages. Images from light microscopy with Hoffman modulation contrast (**a**–**c**) and FSC/SSC characteristics (**d**–**f**) of MDMs cultured with fetal bovine serum (FBS1, (**a**,**d**); FBS2, (**b**,**e**)) or reduced-serum medium (NUS, (**c**,**f**)) are shown with corresponding dot plots (all single cells–gray color, live cells (propidium iodide negative)–red color). Images are representative of at least four pigs. Mean values for FSC-A and SSC-A are marked under each dot plot, including the coefficient of variation (CV). Data represent the mean of 18 MDM cultures for each supplementation.

**Figure 2:**
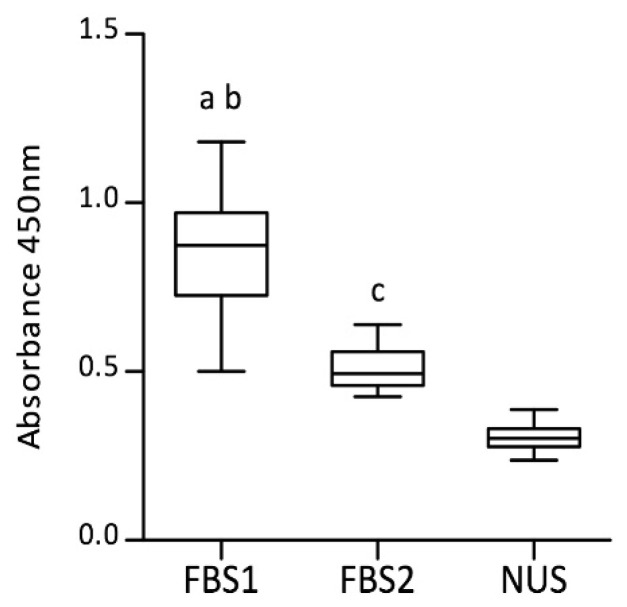
Counts of viable monocyte-derived macrophages. Data obtained using Cell Counting Kit-8 are expressed as absorbance (450 nm) values. Boxplots represent data from the MDM culture with fetal bovine serum (FBS1 and FBS2) or with reduced-serum medium (NUS): median (line), first and third quartiles (box), and minimum and maximum (whiskers), *n* = 18. Significant differences (Friedman test with Dunn’s multiple-comparison post hoc test) between groups are marked as follows: ^a^ FBS1 vs. FBS2, *p* < 0.05; ^b^ FBS1 vs. NUS, *p* < 0.05; ^c^ FBS2 vs. NUS, *p* < 0.05.

Freshly isolated blood monocytes were differentiated into macrophages for 5 days in a D-MEM medium supplemented with 10% FBS (from two different providers) or a reduced-serum medium supplement (NUS). The obtained MDMs presented a unique morphology dependent on culture conditions. D-MEM with 10% of FBS (FBS1 and FDS2) led to mostly round or oval macrophages, whereas the presence of NUS also induced a minor proportion of spindle cells (Figure 1a–c). A similar trend was observed among donors. The different morphology was also confirmed by the flow cytometry parameters (Figure 1d–f). FBS1 culture showed the highest values for both forward scatter (FSC, representing cell size) and side scatter (SSC, representing the internal complexity, i.e., granularity). Significant differences in both the FSC mean value (FBS1 vs. FBS2, *p* < 0.01; FBS1 vs. NUS, *p* < 0.001; FBS2 vs. NUS, *p* > 0.05) and the SSC mean value (FBS1 vs. FBS2, *p* < 0.05; FBS1 vs. NUS, *p* < 0.001; FBS2 vs. NUS, *p* < 0.01) were found when data were evaluated using Friedman test paired with Dunn’s multiple-comparison post hoc test. The coefficient of variation (CV), which determines the cell-to-cell variability within a population, showed there were no statistical significances in FSC between cultures, but the CV parameter for SSC was significantly lower for FBS1 than both FBS2 (*p* < 0.001) and NUS (*p* < 0.05). Lastly, the culture conditions had a significant impact on the macrophage yield. The number of viable MDMs evaluated using Cell Counting Kit-8 (CCK-8) was the highest in D-MEM with 10% FBS1, followed by D-MEM with 10% FBS2 (Figure 2). Significant differences were found between all groups when data were evaluated using Friedman test paired with Dunn’s multiple-comparison post hoc test (FBS1 vs. FBS2, *p* < 0.01; FBS1 vs. NUS, *p* < 0.001; FBS2 vs. NUS, *p* < 0.01). In conclusion, the monocytes were successfully differentiated into macrophages in both FBS-supplemented and reduced-serum conditions, though with variations in yield and morphology.

### 3.2. Surface Marker Expression

**Figure 3:**
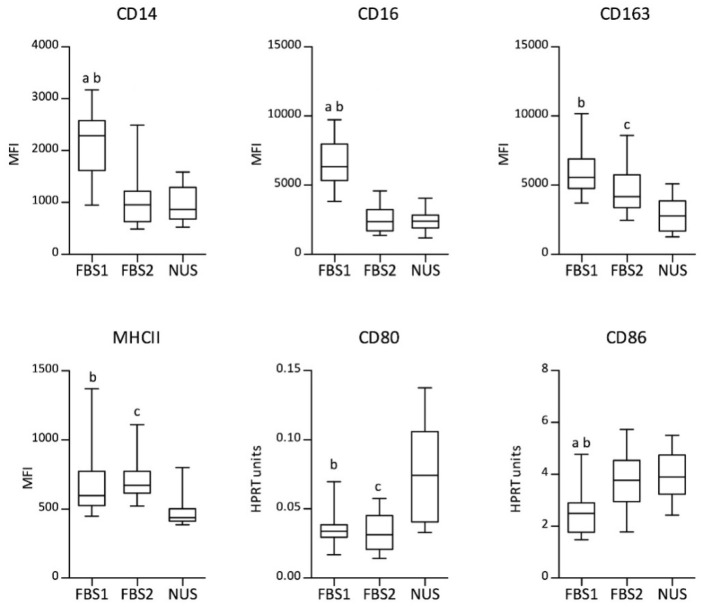
Surface markers CD14, CD16, CD163, and MHCII were assessed by flow cytometry and presented as the median fluorescence intensity (MFI). Relative expressions of CD80 and CD86 measured by qPCR are presented as HPRT units. Boxplots represent data from the MDM culture with fetal bovine serum (FBS1 and FBS2) or with reduced-serum medium (NUS): median (line), first and third quartiles (box), and minimum and maximum (whiskers); flow cytometry: *n* = 18, qPCR: *n* = 12. Significant differences (Friedman test with Dunn’s multiple-comparison post hoc test) between groups are marked as follows: ^a^ FBS1 vs. FBS2, *p* < 0.05; ^b^ FBS1 vs. NUS, *p* < 0.05; ^c^ FBS2 vs. NUS, *p* < 0.05.

Surface markers were assessed by flow cytometry (CD14, CD16, CD163, and MHC II) or qPCR (CD80 and CD86). Flow cytometry data are presented as the median fluorescence intensity (MFI) according to the gating strategy shown in Figure S1. Particular medium supplements had a significant impact on the monitored parameters (Figure 3). Macrophage markers CD14 and CD16 were significantly increased in D-MEM containing FBS1 compared with FBS2 and NUS (paired Friedman test with Dunn’s multiple-comparison post hoc test; *p* < 0.001). This trend accompanied low CD86 mRNA levels (FBS1 vs. FBS2 or NUS, *p* < 0.01). The levels of the scavenger receptor CD163 and MHC class II appeared higher in the presence of serum (both FBS1 and FBS2) than in reduced-serum conditions (CD163: NUS vs. FBS1, *p* < 0.001; NUS vs. FBS2, *p* < 0.01; MHC II: NUS vs. FBS1, *p* < 0.01; NUS vs. FBS2, *p* < 0.001). In contrast, NUS induced a significant increase in CD80 expression at the mRNA level (NUS vs. FBS1, *p* < 0.05; NUS vs. FBS2, *p* < 0.001). In summary, the profiles of the expressed receptors in porcine MDMs significantly differed depending on the medium supplement. Remarkably, differences in the expression level were detected not only between reduced-serum and serum-containing conditions but also between the FBSs from the two providers.

### 3.3. Respiratory Burst and Phagocytic Activity

**Figure 4:**
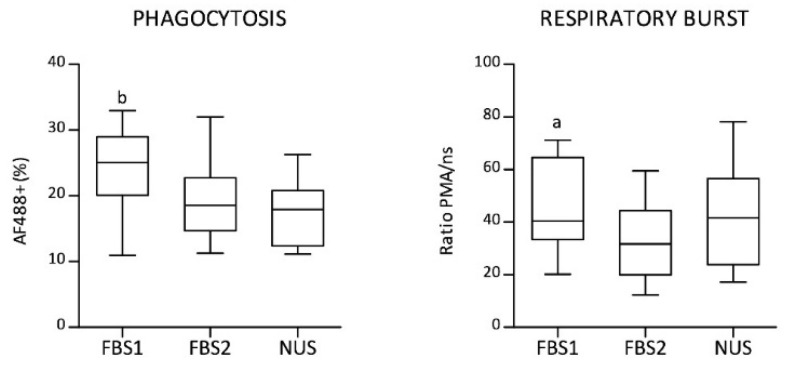
Phagocytic activity and respiratory burst. Phagocytic activity assessed by flow cytometry is presented as a percentage of MDMs positive for Alexa Fluor 488-conjugated (AF488^+^) opsonized zymosan A bioparticles. Respiratory burst measured by a chemiluminescence assay presented as the PMA-stimulated/unstimulated ratio of samples from integrals of chemiluminescence intensity. Boxplots represent data from the MDM culture with fetal bovine serum (FBS1 and FBS2) or with reduced-serum medium (NUS): median (line), first and third quartiles (box), and minimum and maximum (whiskers); *n* = 14 (phagocytosis), *n* = 15 (respiratory burst). Significant differences (Friedman test with Dunn’s multiple-comparison post hoc test) between groups are marked as follows: ^a^ FBS1 vs. FBS2, *p* < 0.05; ^b^ FBS1 vs. NUS, *p* < 0.05.

In order to confirm that reduced-serum culture conditions are suitable for in vitro functional studies of porcine macrophages, their phagocytic activity, respiratory burst, and cytokine production were assessed. Fluorescent-conjugated opsonized zymosan A bioparticles were used for MDM phagocytic activity detection by flow cytometry. Live MDMs were gated according to a gating strategy (Figure S2). About 20% of MDMs were found to be Alexa Fluor 488-positive (Figure 4). The highest proportion of phagocytic cells was found in the FBS1-supplemented MDM culture (median 25.05%), and the difference in comparison with the reduced-serum culture (median 18.55%) was considered significant (*p* < 0.05). The macrophages derived in the presence of FBS2 showed comparable phagocytic activity (median 17.9%) to the reduced-serum culture. Reactive oxygen species (ROS) production was measured after stimulation with PMA using a chemiluminescence assay. The PMA-stimulated/unstimulated ratios of samples were calculated from integrals of chemiluminescence intensity (Figure 4). The ratio values were nearly identical for cultures supplemented with FBS1 (median = 40.4) and NUS (median = 41.6). Nevertheless, a significant difference (*p* < 0.05) was found between FBS1 and FBS2 (median = 31.8) when data were evaluated using Friedman test paired with Dunn’s multiple-comparison post hoc test. Taken together, these data indicate that reduced-serum conditions had no negative impact on macrophage function in terms of respiratory burst and phagocytic activity.

### 3.4. Cytokine Gene Expression Profile

**Figure 5:**
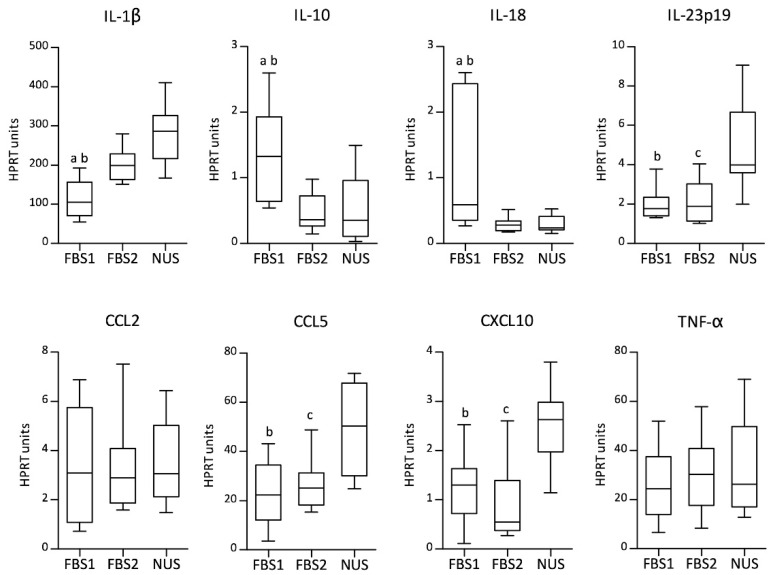
The relative mRNA expression of IL-1β, IL-10, IL-18, IL-23p19, CCL2, CCL5, CXCL10, and TNF-α assessed by qPCR is presented as HPRT units. Boxplots represent data from the MDM culture with fetal bovine serum (FBS1 and FBS2) or with reduced-serum medium (NUS): median (line), first and third quartiles (box), and minimum and maximum (whiskers); *n* = 12. Significant differences (Friedman test with Dunn’s multiple-comparison post hoc test) between groups are marked as follows: ^a^ FBS1 vs. FBS2, *p* < 0.05; ^b^ FBS1 vs. NUS, *p* < 0.05; ^c^ FBS2 vs. NUS, *p* < 0.05.

The mRNA expression levels of the cytokines and chemokines IL-1β, IL-10, IL-18, IL-23p19, CCL2, CCL5, CXCL10, and TNF-α were assessed by qPCR (Figure 5). Cytokine mRNA expression after zymosan A stimulation differed significantly depending on the culture conditions. Macrophages from the FBS-supplemented culture did not share the same cytokine profile when FBS from different providers was used. Significantly higher IL-10 (*p* < 0.05) and IL-18 (*p* < 0.01) levels were found for MDMs with FBS1 (Friedman test with Dunn’s multiple-comparison post hoc test). In contrast, macrophages produced higher levels of IL-1β when cultured with FBS2 rather than FBS1 (*p* < 0.05). MDMs originating from reduced-serum conditions shared some features with FBS2 in contrast to the FBS1-suplemented culture, including significantly lower IL-10 (*p* < 0.01) and IL-18 (*p* < 0.01), but higher IL-1β (*p* < 0.001) mRNA expression. On the other hand, they showed several unique features, including high mRNA expression of proinflammatory cytokine IL-23p19 (NUS vs. FBS1 or FBS2, *p* < 0.01) as well as Th1-related chemokines CXCL10 (NUS vs. FBS1, *p* < 0.01; NUS vs. FBS2, *p* < 0.001) and CCL5 (NUS vs. FBS1, *p* < 0.001; NUS vs. FBS2, *p* < 0.01). The expression of the two remaining genes, TNF-α and CCL2, was similar in MDMs for all medium supplements used in the study. The obtained data confirmed that medium supplementation with NUS or specific FBS leads to MDMs with different cytokine profiles after zymosan A stimulation.

### 3.5. Principal Component Analysis (PCA)

**Figure 6:**
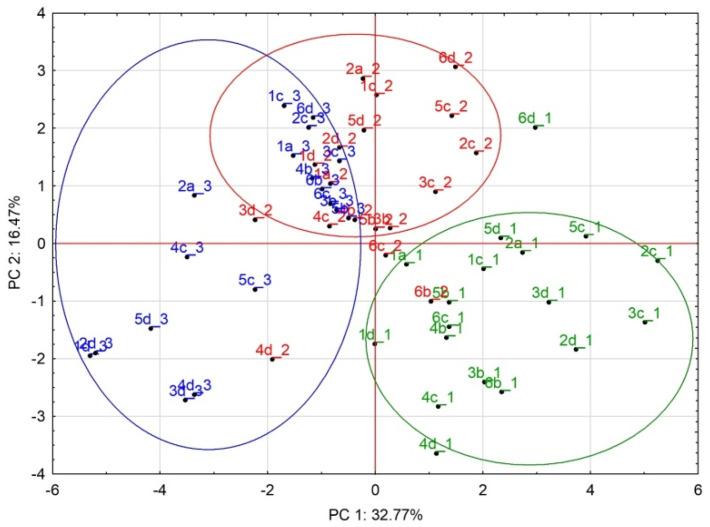
Scatter plot from the principal component analysis (PCA). The analysis included 17 parameters generated from the MDM culture with FBS1 (green, _1), FBS2 (red, _2), or reduced-serum medium (NUS; blue, _3), *n* = 18.

Principal component analysis (PCA) was used to summarize the results of the study. Seventeen parameters generated in this study were analyzed: cell counts assessed by CCK-8, MFI for surface markers (CD14, CD16, CD163, and MHCII), mRNA expression for selected parameters (CD80, DC86, IL-1β, IL-10, IL-18, IL-23p19, CCL2, CCL5, CXCL10, and TNF-α), percentage of phagocytic MDMs, and data from a chemiluminescence assay (PMA-stimulated/unstimulated ratios from integrals of chemiluminescence intensity). It can be seen in the scatter plot (Figure 6) that the analyzed data clearly differentiate into three separate identifiable clusters in the coordinate system of the first two principal components (PC1 and PC2). These first two principal components explain approximately 49.23% of the variability in the measured data. In particular, a well-separated cluster for FBS1 shows a statistically significant similarity in the results of the analyzed parameters. PCA confirmed the significant impact of culture conditions on MDM characteristics. Surprisingly, the difference between both FBSs was much more pronounced than the effect of the reduced-serum supplement. While MDMs from the FBS1 and FBS2 culture gave rise to two separate clusters, MDMs cultured in the presence of FBS2 and NUS showed partial clustering.

## 4. Discussion

The aim of this study was to provide an improved in vitro model of porcine monocyte-derived macrophages based on a reduced-serum culture. A reduction in or complete absence of serum is suitable for experimental studies focused on molecules naturally present in serum or in cultures used for subsequent protein purification. Moreover, the benefit of serum-free or reduced-serum supplements follows the current trend of reducing FBS use in biological research. In this study, macrophages were derived from enriched CD14^+^ blood monocytes during 5-day cultivation. MDM characteristics were compared depending on their culture in reduced-serum medium supplement (NUS) vs. conventional FBS-based culture. We found a significant impact of culture conditions used in the study, including in terms of MDM morphology, yield, surface marker expression, and cytokine profiles. Phagocytic and respiratory burst capacity were not negatively affected by FBS reduction. FBS contains a broad spectrum of cell growth-supporting macromolecules (growth factors, proteins, lipids, carbohydrates, hormones, and enzymes), and it is the most commonly used additive. As an example of its influence, 10% of FBS medium usually contains 1–2 ng/mL total TGF-β [22] while the equivalent concentration of TGF-β induces a dramatic change in macrophage morphology and decreases NO production and phagocytic capacity [23]. The impact on MDM morphology was clearly documented in this study, where different FBSs were used for porcine MDM differentiation. One of the features of macrophage differentiation is enhanced granularity, and that cells become significantly larger with time, as demonstrated by the increase in side scatter (SSC) and forward scatter (FSC) in flow cytometry [24,25]. On the basis of these parameters, FBS1-supplemented cells seem to represent a more mature MDM culture when compared with FBS2- and NUS-supplemented cells. Macrophage culture supplemented with FBS1 also gave rise to macrophages with a higher level of CD14, CD16 (FcRIII), and CD163, which are markers of macrophage differentiation. The expression of each marker is induced over time in pig bone marrow-derived macrophage cultures [26]. In contrast, porcine CD14 antigen is downregulated as monocytic cells differentiated into macrophages, both in vitro and in vivo [25]. Additionally, the higher IL-10 and lower IL-1β mRNA levels found in FBS1 culture suggests possible anti-inflammatory properties. However, time-dependent differentiation or macrophage polarization were not the aims of the study. The main aim of our study was to establish a method for serum-reduced MDM culture with an emphasis on affordable costs. For this reason, cytokines or growth factors were not included in the protocol. The obtained MDMs showed several common features with the FBS2-supplemented culture. Given the significant differences resulting from the use of FBS from two different providers in the study, reduced-serum culture offers an acceptable alternative for porcine MDM culture. Increased data reproducibility across specific studies and support of the current trend to reduce FBS use are indisputable advantages of MDM cultivation in reduced-serum conditions. In addition, a number of serum-reduced or serum-free medium formulations have been described for cell lines as well as for primary cells [13,27]. Such formulations have been successfully established for human MDMs [28], murine bone marrow-derived macrophages [29], and porcine bone marrow-derived M1- and M2-polarized macrophages [10]. However, recombinant growth factors or cytokines were used in the abovementioned studies, which increased the protocol price. The Nu-Serum™ Growth Medium Supplement offers an affordable compromise with a reduced FBS concentration in the culture. We demonstrated that the reduced-serum conditions were suitable for in vitro generation of porcine MDMs. The obtained MDMs showed increased mRNA levels of IL-1b and CD86 as well as proinflammatory cytokines, including IL-23p19, CXCL10, and CCL5. A disadvantage of the reduced-serum culture was the lower yield of viable MDMs, in accordance with previously published data on murine bone marrow macrophages [30]. In addition, a lower surface expression of scavenger receptor CD163 was found on MDMs from the NUS culture. This should be particularly taken into account in experiments dealing with porcine reproductive and respiratory syndrome virus (PRRSV), because CD163 is an essential receptor for virus infection [31,32].

## 5. Conclusions

In conclusion, our study confirmed that the selection of medium supplements can influence the immunomodulation of in vitro-generated porcine MDMs. It is necessary to keep in the mind that MDMs still represent an in vitro model, and it is crucial to respect the requirements of particular experiments or hypotheses when using the model. Three particular aspects of the MDM culture protocol established here can be highlighted: (1) use of a serum-reduced medium; (2) obtention of macrophages with functional biological properties; (3) cost-effective protocol. Reduced-serum culture is a useful tool for the generation of porcine MDMs as an alternative to conventional FBS supplementation. The search for additional affordable serum-free medium supplement alternatives will continue to be a challenge for future research in this field.

## Data Availability

Not applicable.

## Supplementary Materials

The following supporting information can be downloaded at https://www.mdpi.com/article/10.3390/biology11101457/s1: Figure S1. Gating strategy—surface markers; Figure S2. Gating strategy—phagocytosis.

## References

[1] Wynn T.A., Chawla A., Pollard J.W. (2013). Macrophage Biology in Development, Homeostasis and Disease. Nature.

[2] Taciak B., Białasek M., Braniewska A., Sas Z., Sawicka P., Kiraga Ł., Rygiel T., Król M. (2018). Evaluation of Phenotypic and Functional Stability of RAW 264.7 Cell Line through Serial Passages. PLoS ONE.

[3] Kyrova K., Stepanova H., Rychlik I., Polansky O., Leva L., Sekelova Z., Faldyna M., Volf J. (2014). The Response of Porcine Monocyte Derived Macrophages and Dendritic Cells to Salmonella Typhimurium and Lipopolysaccharide. BMC Vet. Res..

[4] Stepanova H., Pavlova B., Stromerova N., Ondrackova P., Stejskal K., Slana I., Zdrahal Z., Pavlik I., Faldyna M. (2012). Different Immune Response of Pigs to Mycobacterium Avium Subsp. Avium and Mycobacterium Avium Subsp. Hominissuis Infection. Vet. Microbiol..

[5] Kavanová L., Matiašková K., Levá L., Nedbalcová K., Matiašovic J., Faldyna M., Salát J. (2018). Concurrent Infection of Monocyte-Derived Macrophages with Porcine Reproductive and Respiratory Syndrome Virus and Haemophilus Parasuis: A Role of IFNα in Pathogenesis of Co-Infections. Vet. Microbiol..

[6] Kavanová L., Moutelíková R., Prodělalová J., Faldyna M., Toman M., Salát J. (2018). Monocyte Derived Macrophages as an Appropriate Model for Porcine Cytomegalovirus Immunobiology Studies. Vet. Immunol. Immunopathol..

[7] Kavanová L., Matiašková K., Levá L., Štěpánová H., Nedbalcová K., Matiašovic J., Faldyna M., Salát J. (2017). Concurrent Infection with Porcine Reproductive and Respiratory Syndrome Virus and Haemophilus Parasuis in Two Types of Porcine Macrophages: Apoptosis, Production of ROS and Formation of Multinucleated Giant Cells. Vet. Res..

[8] Vicenova M., Nechvatalova K., Chlebova K., Kucerova Z., Leva L., Stepanova H., Faldyna M. (2014). Evaluation of in Vitro and in Vivo Anti-Inflammatory Activity of Biologically Active Phospholipids with Anti-Neoplastic Potential in Porcine Model. BMC Complement. Altern. Med..

[9] Zemankova N., Chlebova K., Matiasovic J., Prodelalova J., Gebauer J., Faldyna M. (2016). Bovine Lactoferrin Free of Lipopolysaccharide Can Induce a Proinflammatory Response of Macrophages. BMC Vet. Res..

[10] Gao J., Scheenstra M.R., van Dijk A., Veldhuizen E.J.A., Haagsman H.P. (2018). A New and Efficient Culture Method for Porcine Bone Marrow-Derived M1- and M2-Polarized Macrophages. Vet. Immunol. Immunopathol..

[11] Chamorro S., Revilla C., Álvarez B., Alonso F., Ezquerra Á., Domínguez J. (2005). Phenotypic and Functional Heterogeneity of Porcine Blood Monocytes and Its Relation with Maturation. Immunology.

[12] Franzoni G., Bonelli P., Graham S.P., Anfossi A.G., Dei Giudici S., Pilo G., Pittau M., Nicolussi P., Oggiano A. (2017). Comparative Phenotypic and Functional Analyses of the Effects of Autologous Plasma and Recombinant Human Macrophage-Colony Stimulating Factor (M-CSF) on Porcine Monocyte to Macrophage Differentiation. Vet. Immunol. Immunopathol..

[13] van der Valk J., Bieback K., Buta C., Cochrane B., Dirks W.G., Fu J., Hickman J.J., Hohensee C., Kolar R., Liebsch M. (2018). Fetal Bovine Serum (FBS): Past–Present–Future. ALTEX.

[14] Ham R.G. (1965). Clonal Growth of Mammalian Cells In A Chemically Defined, Synthetic Medium. Proc. Natl. Acad. Sci. USA.

[15] Gstraunthaler G. (2003). Alternatives to the Use of Fetal Bovine Serum: Serum-Free Cell Culture. ALTEX Altern. Anim. Exp..

[16] Pavlova B., Volf J., Ondrackova P., Matiasovic J., Stepanova H., Crhanova M., Karasova D., Faldyna M., Rychlik I. (2011). SPI-1-Encoded Type III Secretion System of Salmonella Enterica Is Required for the Suppression of Porcine Alveolar Macrophage Cytokine Expression. Vet. Res..

[17] Kyrova K., Stepanova H., Rychlik I., Faldyna M., Volf J. (2012). SPI-1 Encoded Genes of Salmonella Typhimurium Influence Differential Polarization of Porcine Alveolar Macrophages in Vitro. BMC Vet. Res..

[18] Volf J., Boyen F., Faldyna M., Pavlova B., Navratilova J., Rychlik I. (2007). Cytokine Response of Porcine Cell Lines to Salmonella Enterica Serovar Typhimurium and Its HilA and SsrA Mutants. Zoonoses Public Health.

[19] Andersen C.L., Jensen J.L., Ørntoft T.F. (2004). Normalization of Real-Time Quantitative Reverse Transcription-PCR Data: A Model-Based Variance Estimation Approach to Identify Genes Suited for Normalization, Applied to Bladder and Colon Cancer Data Sets. Cancer Res..

[20] Bustin S.A., Benes V., Garson J.A., Hellemans J., Huggett J., Kubista M., Mueller R., Nolan T., Pfaffl M.W., Shipley G.L. (2009). The MIQE Guidelines: Minimum Information for Publication of Quantitative Real-Time PCR Experiments. Clin. Chem..

[21] Livak K.J., Schmittgen T.D. (2001). Analysis of Relative Gene Expression Data Using Real-Time Quantitative PCR and the 2-ΔΔCT Method. Methods.

[22] Oida T., Weiner H.L. (2010). Depletion of TGF-β from Fetal Bovine Serum. J. Immunol. Methods.

[23] Mills C.D., Kincaid K., Alt J.M., Heilman M.J., Hill A.M. (2000). M-1/M-2 Macrophages and the Th1/Th2 Paradigm. J. Immunol..

[24] Daigneault M., Preston J.A., Marriott H.M., Whyte M.K.B., Dockrell D.H. (2010). The Identification of Markers of Macrophage Differentiation in PMA-Stimulated THP-1 Cells and Monocyte-Derived Macrophages. PLoS ONE.

[25] McCullough K.C., Basta S., Knötig S., Gerber H., Schaffner R., Kim Y.B., Saalmüller A., Summerfield A. (1999). Intermediate Stages in Monocyte-Macrophage Differentiation Modulate Phenotype and Susceptibility to Virus Infection. Immunology.

[26] Kapetanovic R., Fairbairn L., Beraldi D., Sester D.P., Archibald A.L., Tuggle C.K., Hume D.A. (2012). Pig Bone Marrow-Derived Macrophages Resemble Human Macrophages in Their Response to Bacterial Lipopolysaccharide. J. Immunol..

[27] Brunner D., Frank J., Appl H., Schöffl H., Pfaller W., Gstraunthaler G. (2010). Serum-Free Cell Culture: The Serum-Free Media Interactive Online Database. ALTEX Altern. Anim. Exp..

[28] Rey-Giraud F., Hafner M., Ries C.H. (2012). In Vitro Generation of Monocyte-Derived Macrophages under Serum-Free Conditions Improves Their Tumor Promoting Functions. PLoS ONE.

[29] Eske K., Breitbach K., Köhler J., Wongprompitak P., Steinmetz I. (2009). Generation of Murine Bone Marrow Derived Macrophages in a Standardised Serum-Free Cell Culture System. J. Immunol. Methods.

[30] Flesch I., Ferber E. (1986). Growth Requirements of Murine Bone Marrow Macrophages in Serum-Free Cell Culture. Immunobiology.

[31] Calvert J.G., Slade D.E., Shields S.L., Jolie R., Mannan R.M., Ankenbauer R.G., Welch S.-K.W. (2007). CD163 Expression Confers Susceptibility to Porcine Reproductive and Respiratory Syndrome Viruses. J. Virol..

[32] Patton J.B., Rowland R.R., Yoo D., Chang K.O. (2009). Modulation of CD163 Receptor Expression and Replication of Porcine Reproductive and Respiratory Syndrome Virus in Porcine Macrophages. Virus Res..

