# Supplementary material for: Characterization of Porcine Monocyte-Derived Macrophages Cultured in Serum-Reduced Medium
Source: Biology (Basel). 2022 Oct 4;11(10):1457. doi: 10.3390/biology11101457 (PMC9598231; doi:10.3390/biology11101457)

Figure S1. Gating strategy – surface markers. Surface markers CD14, CD16, CD163 and MHCII were assessed by flow cytometry and presented as median of fluorescence intensity (MFI). Representative dotplots from one MDM culture (10% FBS1) are included in the gating strategy.

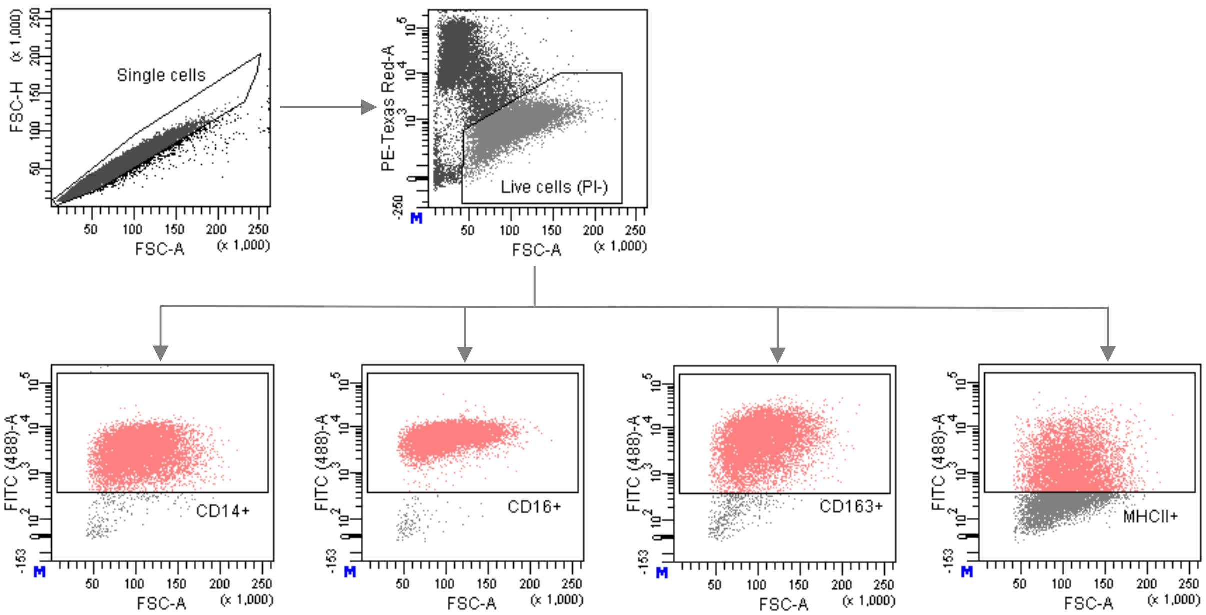

Supplement: Supplementary file 1 [file biology-11-01457-s001.zip › Figure S1-supplementary.pdf]
