# Supplementary material for: Characterization of Porcine Monocyte-Derived Macrophages Cultured in Serum-Reduced Medium
Source: Biology (Basel). 2022 Oct 4;11(10):1457. doi: 10.3390/biology11101457 (PMC9598231; doi:10.3390/biology11101457)

Figure S2. Gating strategy – phagocytosis. Phagocytic activity assessed by flow cytometry is presented as percentage of MDM positive for Alexa-fluor 488-conjugated (Zymozan+) opsonized zymozan A bioparticles. Representative dotplots from one MDM culture (10% FBS1) is included in the gating strategy.

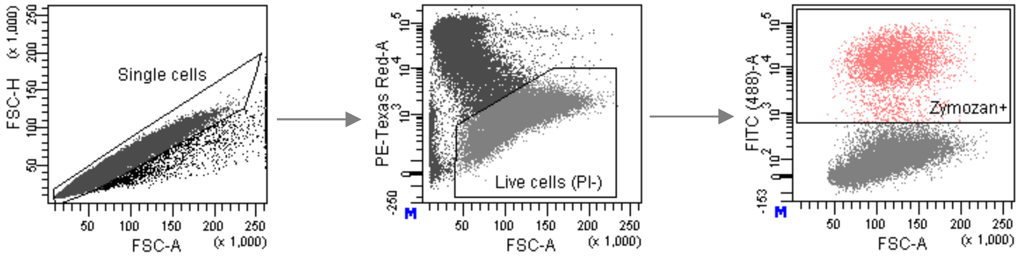

Supplement: Supplementary file 1 [file biology-11-01457-s001.zip › Figure S2_supplementary.pdf]
